# *‘I didn't know what was in front of me’*—Bereaved parents' experience of adapting to life when a co‐parent of dependent children has died with cancer

**DOI:** 10.1002/pon.6010

**Published:** 2022-09-05

**Authors:** Jeffrey R. Hanna, Cherith J. Semple

**Affiliations:** ^1^ School of Nursing Ulster University Newtownabbey UK; ^2^ South Eastern Health and Social Care Trust Cancer Services Ulster Hospital Belfast UK

**Keywords:** bereavement, cancer, death, dependent children, oncology, parental cancer, parents, psychosocial support, qualitative research

## Abstract

**Objective:**

It is not clear how the bereaved parent navigates life with the children after a co‐parent has died. The objective of this study is to explore bereaved parents' experience of managing life when a co‐parent of dependent children has died with cancer.

**Methods:**

Twenty‐one in‐depth interviews were conducted with bereaved parents when a co‐parent died with cancer. Data were analysed thematically.

**Results:**

Bereaved parents highlighted how their children was their key focus after the death of a co‐parent with cancer, as they effortfully strived to be a ‘perfect parent’. While some bereaved parents struggled to adapt to the role as a sole parent, others described the importance of maximising social networks to help with the practical aspects of parenting. However, most bereaved parents described intense feelings of loneliness as they navigated parenting alone. To help navigate this post‐bereavement period, parents considered it important for their children to openly talk about the deceased parent. Also, meeting others who have experienced similar situations was helpful for the bereaved parent and children, providing hope for the future. Results are discussed under two themes: (1) adapting to life without the parent, and (2) a desire to keep the memory alive of the parent that died with cancer.

**Conclusions:**

Bereaved parents should be encouraged to practice self‐care when a co‐parent has died from cancer so they can appropriately meet the needs of their children. Bereaved parents should be reassured that showing emotion in front of the children is helpful and could facilitate better grief experiences for the whole family.

## 1 BACKGROUND

When a parent of dependent children (<18 years old, hereafter referred to as children) has an incurable cancer prognosis, the illness is often ‘normalised’ within the family; routine continues with an ever‐changing availability of parenting from both parents to the final weeks and days of life.^1^, ^2^ Many parents require advice and guidance from health and social care professionals toward preparing children for the end of life experience of a parent with cancer.^3^, ^4^ The immediate post death period is an exceptionally challenging time for the newly bereaved parent regarding the children, with some studies suggesting the funeral director can have an important role in guiding the bereaved parent through this period.^5^, ^6^ There is a clear lack of understanding as to how bereaved parents cope and manage life with the children beyond this juncture.

Bereavement is often a period of major transition for individuals and can involve disruption to a person's routine, available social networks and relationships.^7^, ^8^, ^9^ When a parent has died, the bereaved parent is now having to navigate their own grief and the children's, whilst managing parenting alone.^10^, ^11^ Sadly, this is not an uncommon phenomenon for families, with 20% of children in the United Kingdom experiencing the death of a parent before their eighteenth birthday.^12^ Parental death in childhood predisposes children to increased levels of anxiety, depression, risk‐taking behaviours and offending, as well as lower academic attainment than the general population.^13^, ^14^, ^15^ While parental support can help mediate such adversities,^16^, ^17^ it is not clear how bereaved parents are supporting the children when a parent has died with cancer, who are also at risk of adverse mental health outcomes.^18^, ^19^ Investigating how bereaved parents navigate bereavement when a co‐parent has died with cancer could aid our understanding if, how and when they can be supported as they care for their children.

### 1.1 Aims and objectives

The aim of this study is to explore bereaved parents' experience of managing family life when a co‐parent of dependent children has died with cancer. The objectives are to investigate:

(i) how bereaved parents navigated family life when a co‐parent of dependent children has died with cancer,
(ii) how bereaved parents supported their children when a co‐parent of dependent children has died with cancer,
(iii) what bereaved parents consider as a good practice in bereavement when a co‐parent of dependent children has died with cancer.

## 2 METHODS

This study is a qualitative design using in‐depth interviews. This approach is most appropriate when exploring individual experiences. This study is reported following the Consolidated Criteria for Reporting Qualitative Research guidelines.^20^

### 2.1 Study population

Individuals were considered eligible if they were a parent of dependent children (<18 years old) and had experienced the death of a co‐parent to cancer. There were no limits applied to a minimum period between death and inclusion to the study to promote an individual's autonomy toward taking part.^21^

### 2.2 Sampling

Convenience and volunteer sampling techniques identified bereaved parents from the general public, a hospice and a family support service throughout Northern Ireland. It is worthwhile to note that the family support service provides pre‐and‐post bereavement support to families impacted by parental cancer and sits within a cancer charity.

### 2.3 Recruitment

A family support worker at the support service and a social worker at the hospice identified bereaved parents against the inclusion criteria from their databases. Those eligible were directly mailed information about the purpose of the study and what would be involved, with no further introduction, explanation or follow‐up. Due to data protection measures of storing and handling personal information, both sites could only invite those parents who were bereaved within the last 5 years. A total of 18 out of 35 eligible individuals did not respond to the study invitation. Of the 17 individuals that expressed interest in taking part, ninewere currently receiving support from the hospice or the family support service.

To promote study accrual and representation of families beyond support groups and hospice services, a public advert was developed by the research team and steering group. This was published on three occasions in a national newspaper and displayed in 183 public spaces including clinical settings, community and leisure centres and supermarkets. A total of four eligible individuals expressed interest and took part. Written and verbal consent was obtained at the time of interview.

Study recruitment was open between January 2018 and February 2020.

### 2.4 Data collection

Face‐to‐face in‐depth interviews were conducted between April 2018 and February 2020. A topic guide with open‐ended questions was developed informed by the literature, study aims and objectives, alongside the research team and steering group. The steering group consisted of a family support worker, a social worker, a palliative care specialist nurse as well as a bereaved parent and teenager. The guide was iteratively modified throughout the data collection period to ensure follow‐up with categories in subsequent interviews (Table 1). Interviews were conducted by both authors; CJS is a female cancer nurse specialist with a wealth of qualitative research experience, and JRH is a male researcher with experience of qualitative research at end of life and bereavement. The researchers were not known to the participants. Interviews were audio‐recorded and last between 60 and 120 min at a venue convenient for participants (home [*n* = 16], work [*n* = 2], support service site [*n* = 3]). Interviews were completed when no further categories were identified by the researchers [JRH, CJS].

**TABLE 1:** Topic guide used to guide the conduct of the study

**Initial topic guide based on the literature, research aims and objectives and expert group.**
Explore bereaved parents' experiences of family life following the death of a co‐parent to cancer Explore bereaved parents' experiences of navigating life with the children when a co‐parent has died with cancer Explore bereaved parents' perceptions of what helped them toward managing life when a co‐parent died with cancer
**Sample of additional topics following identification of initial categories.**
4. The role of peer‐support when a co‐parent has died with cancer. 5. Experiences of loneliness with a co‐parent has died with cancer. 6. The role of social networks to help with parenting when a co‐parent has died with cancer.

### 2.5 Data analysis

Audio‐recordings were transcribed verbatim and verified by the research team. The data was analysed using reflexive thematic analysis; a flexible method useful to exploring individual experiences.^22^, ^23^ Initially, JRH read and reread the transcripts to gain a sense of each bereaved parent's story. Then, JRH coded the data, detailing inductive descriptive codes by marking similar phrases or words from the participant's narratives using Microsoft Word. Reflexive thematic analysis was a useful approach to enabling JRH to reflect and engage with the data, generating themes from the codes using mind mapping techniques.^23^, ^24^ Initially JRH identified three themes, however through discussion and refinement as a research team (JRH, CJS) some of the data overlapped between two of these themes, and were subsequently merged to one theme. Feedback was obtained from a PPI representative.

### 2.6 Ethical considerations

Individuals were not coerced to take part and were informed of their right to withdraw at any time, and this would not impact on any relationship with the organisation they may have been identified from. A distress protocol was established. The research team acknowledge that talking about issues such as parental cancer and bereavement may trigger reactions for participants, therefore an information and support pack was provided as part of the debriefing process which included the contact details of organisations that offer support for families following a parental death to cancer. Given the sensitive nature of the research topic, opportunities for reflection and debriefing were provided throughout the study for the research team. Data protection procedures were observed and assurance of confidentiality were given. Pseudonyms have been applied throughout the results section. Ethical approvals were obtained at institutional and national levels [REC:17/SW/0155].

## 3 RESULTS

A total of 21 bereaved parents took part in the study; 12 were mothers and nine were fathers. At the time of interview, bereaved parents were between 5 weeks and 6 years (*mAverage = 14.6 months; median = 10.5 months*) after the death of the parent that died with cancer. Bereaved parents were recruited from a family support service (*n* = 14), a hospice (*n* = 3) and a public advert (*n* = 4). Sample characteristics are reported in Table 2.

**TABLE 2:** Sample characteristics of the 21 bereaved parents included in the study

Variable	N (%)
Participant (bereaved parents)	
Bereaved father	9 (42.86)
Bereaved mother	12 (57.14)
Cancer site	
Pancreatic	2 (9.52)
Lung	1 (4.76)
Bowel	1 (4.76)
Breast	2 (9.52)
Head and neck	1 (4.76)
Glioblastoma	3 (14.3)
Melanoma	2 (9.52)
Renal	1 (4.76)
Oesophageal	3 (14.3)
Angiosarcoma	1 (4.76)
Liver	2 (9.52)
Ovarian	2 (9.52)
Marital status at time of death	
Married	21 (100)
Gender/age of children at time of death	
Boy, 0–11 years old	15 (71.43)
Boy, 12–18 years old	7 (33.33)
Girl, 0–11 years old	19 (90.48)
Girl, 12–18 years old	12 (57.12)
Ethnicity of bereaved parent	
White	20 (95.24)
Asian	1 (4.76)
Recruitment source	
Hospice service	3 (14.3)
Public advert	4 (19.04)
Family support service	14 (66.66)
Place of death	
Home	5 (23.81)
Hospice	7 (33.33)
Hospital	9 (42.86)
Socioeconomic details	
Highest level of completed education	
Secondary level (GCSE/A‐Level)	6 (28.57)
Skills based qualification	2 (9.52)
Bachelor's degree	11 (52.39)
Master's degree	2 (9.52)
Employment status at time of death	
Full‐time work	5 (23.81)
Part‐time work	2 (9.52)
Leave of absence	10 (47.61)
Maternity leave	1 (4.76)
Unemployed	3 (14.3)
Main source of household income at time of death	
Employment	7 (33.33)
Statutory sick pay	9 (42.86)
Statutory maternity pay	1 (4.76)
Social security funding	4 (19.05)

The data below are reflective of the first 18 months after the funeral of the parent that died with cancer. Within a Northern Irish context, usually the funeral takes place 2–3 days after the death. The funeral period was not a key focus of this research but has been reported through the lens of funeral directors in another study by the research team.^5^ Results for this study are discussed under two themes: (1) adapting to life without the parent, and (2) a desire to keep the memory alive of the parent that died with cancer.

### 3.1 Theme 1: Adapting to life without the parent

Bereaved parents reported their pain and upset regarding the death of a partner with cancer and being a sole parent of dependent children, often coined as *‘not how we planned our life together’.* However, bereaved parents highlighted their priority after the death of a co‐parent with cancer was for the children. Reflecting on their experience, bereaved parents reported factors that were perceived helpful to them as they navigated family life when a co‐parent of dependent children has died with cancer. These are discussed under two sub‐themes: (i) adjusting to life as a bereaved parent, and (ii) navigating the grief experience as a family.

### 3.2 Sub‐theme 1: Adjusting to life as a bereaved parent

Bereaved parents considered it helpful to continue with the children's routine soon after the funeral to provide security and stability for the whole family. This included the children going back to school and being part of their usual extracurricular activities. At this juncture, it seemed some parents struggled to adjust to the role as a sole parent and taking on the aspects of parenting that had predominately been led by the other parent before they died with cancer. This included the morning routine in the home such as getting the children ready for school.

I was getting up in the morning and leaving at half seven and coming back at half six. And everything with the girls was taken care for in between by Ciara [parent that died]. Fast forward all that, I didn’t know what was in front of me. You were in the position of going right, ‘how do you tell the difference between skirts, jumpers, tights, uniforms’. And you’re being asked to do ponytails for school. I didn’t have a notion as to what I was doing.[Michael, bereaved father]

Bereaved parents expended much energy and focus, described as a desire to ‘*get it right’* for the children after the death of a co‐parent with cancer. For bereaved parents this was striving to maintain the aspects of everyday life that were typical of the family before the cancer, and where both parents were able to help with the practical aspects of parenting. This included looking after the children and taking them to their after‐school clubs. On occasion, bereaved parents felt they had to be a *‘perfect parent’* for the children to make up for what the parent who died can no longer provide. More often, this placed significant pressures on bereaved parents who described feeling overwhelmed at the situation, feeling they had a lack of time for themselves with *‘all the running about’.*

I initially was always trying to do things the way Nicole would try to do them. Nicole would always have been so maternal, where if the kids had a problem, they would always run to their mummy. If I was a painter, I’d have been a big brush, whereas Nicole came along, and she was the fine brush. I was always worried that I’d never be able to match up to her. So, I just became so consumed by making sure everything was perfect.[Peter, bereaved father]

After the death of a co‐parent with cancer there appeared to be a tension for bereaved parents in relation to *‘getting on with life’*. While some parents returned to work soon after the funeral due to financial reasons or working arrangements, others felt they needed more time to adjust to new ways of being a sole parent before going back to work. Bereaved parents highlighted their desire to continue having ‘*fun’* together as a family such as having a day out with the children, but were unsure when this would be appropriate or how this might be viewed by others.

Don’t get me wrong, of course I miss Simon [parent that died]. He was my husband and the father of my children. But I’m really struggling with finding that balance of moving on. I still want my children to have good memories in their childhood despite the fact their daddy died.[Tracey, bereaved mother]

Although not always available for families, it was reported by bereaved parents as helpful to maximise social networks to help with the practical aspects of parenting, to include grandparents, aunts, uncles or older children in the family (18–25 years). This was especially important for bereaved parents as they went back to work, and involved taking the children to and from school, as well as household chores such as cooking and cleaning. Bereaved parents reflected this reduced the demands facing them and facilitated quality time as a family in the evenings and at weekends.

It was different before Gary died. He was at home all the time. He was here when Emma got home from school. I didn’t have to worry about that. But it’s different now. I’m self‐employed so I had to go back to work basically right away. Thankfully I have lots of people to help. She [child] goes to my parents on a Monday and Tuesday. Then my sister takes her on a Wednesday, an after‐school club on Thursday and Gary’s mum has her on a Friday. I don’t know how I’d manage without them.[Mona, bereaved mother]

Bereaved parents reported experiencing intense feelings of loneliness as they were now having to navigate the responsibility of parenting predominately alone. This was evident for bereaved parents as important decisions were being made to include selecting a secondary school for the children, or managing certain periods such as when the younger children (<12 years old) emerged into adolescence.

When you have got someone with you for fifteen years and you make every decision together, do everything together, all of a sudden you are the one that is now, you know, it is horrible.[Claire, bereaved mother]

As time moved on, loneliness was further experienced by bereaved parents with older children (>13 years old) who often reported being home alone, as their teenage children spent more time with friends. There was a strong *‘sense of missing’* in life from bereaved parents in activities that were previously cherished as a couple such as going on holidays or having a meal together.

Joel really loved my cooking. Not that I’m sure I’m any good at it. I guess the fact that he just appreciated everything. It’s just things like that. And the fact it’s such a great summer and he would have loved that, and we would have walked together a lot.[Janice, bereaved mother]

### 3.3 Sub‐theme 2: Navigating the grief experience as a family

Grief was an individualised experience between and within families. The reactions of the children varied, who were often described by bereaved parents as *‘dipping in and out’* of grief. This included times of sadness, upset and anger about the fact their parent died with cancer, parallel with simply living in the moment of everyday ordinariness. Often, bereaved parents felt the children had not processed the reality their parent had died with cancer because they had not grieved in the expected way.

They’ve [children] just got on with things. There’s never any kind of emotional outburst. It’s just mad. It just floors me. It’s working so well that it’s actually suspicious. You’re just thinking that there has to be a downside somewhere.[Ian, bereaved father]

Some bereaved parents reported situations where there were changes to the children's behaviour or traits, coined as *‘unusually characteristic’*. This included displays of aggression at home or not doing as well in their schoolwork. Although these situations were rare, bereaved parents often questioned if such changes were part of being a child or teenager or an element of grief. Also, if they (the parents) had done the *‘right thing’* with the children in the end of life period and sufficiently prepared them for the death of their parent with cancer.

A few weeks ago he cracked up because Carl [sibling] knocked over his Lego. Before he would have been laid‐back over it. It was a storm in a cup. I don’t know if that’s a control thing now that his daddy isn’t here. It’s very difficult. Then I think did Jack [parent that died] and I get it right for the boys before he died. At the time I felt we did, but I don’t know now[Lisa, bereaved mother]

When a co‐parent died with cancer, bereaved parents described the emotional burden of navigating the children through their grief, as well as their own acute grief. Many bereaved parents believed they could not tell the children how they were feeling about the situation as they considered it to be too difficult for the children to see the bereaved parent upset. Fundamentally, bereaved parents felt they had to *‘keep it together’* for the children. For those bereaved parents that did openly share their emotions with the children they perceived it facilitated opportunities for the children to tell the bereaved parent how they were feeling about the situation.

The thing that’s hard to process is how to go through the grieving process. Like, for me, I just do not even go there, because it would just spiral into too much sadness. I need to be strong for the girls.[Zelda, bereaved mother]

There were a few bereaved parents that felt it was useful to access counselling or support services to talk about their grief experience. Conversely, most bereaved parents highlighted it as unhelpful to be encouraged by other close family members that the children or themselves (the bereaved parent) must attend counselling to counteract *‘falling apart’* regarding the death of a co‐parent with cancer.

After Una died you had all these people, who meant well, going ‘okay so when are you going for counseling and when are the kids going to counseling’. You almost get this sense that if you’re not then you must be doing something wrong. They were like you can’t carry all this burden on your shoulders and become a single dad and bring up your kids and deal with your wife’s bereavement.[Ian, bereaved father]

Facilitated by a family support service, bereaved parents stated it was useful to meet others who have experienced similar situations. This included hearing the *‘stories’* of other bereaved families and gleaning tips as to how they navigated elements of life such as going on the first holiday without the co‐parent who had died. Also, bereaved parents considered it important for the children to meet and know there are other young people who have experienced the death of a parent to cancer. Overwhelmingly, bereaved parents believed meeting other families who were further along in the bereavement experience provided hope for the here and now and the future. Most families seemed to stop attending the family support service around 18–20 months after the death of a parent with cancer, as they believed they were *‘not in the same place’* as some of the new families who started to attend.

It was just nice to be able to sit with people who are in the same boat as you and the kids went off to another room and obviously all the kids are in the same boat. For me to know there are other’s going through your same pain and understanding how they’re approaching their day to day. It was just good to hear how they’re dealing with it and how you can maybe handle things differently as well.[Robert, bereaved father]

It was important for **most** bereaved parents to be reassured that the school would update them if there were any concerns about the children. Also, when teachers regularly ‘*checked‐in’* with the children about how they were coping with the situation was reported to provide security for the children and comfort for the bereaved parent. Some bereaved parents felt it would have been supportive for the children if the school prepared the younger children's class before returning to school after the funeral, and explaining to them what it meant to die.

I didn’t know how it would affect them going back into school. Especially the younger two. How would a child tell another child that their parent isn’t coming back? I don’t really think teachers are prepared to deal with grief in the classroom. It was me dealing with the fallout of it when they [children] came home crying in the evening.[Emma, bereaved mother]

### 3.4 Theme 2: A desire to keep the memory alive of the parent that died with cancer

Talking about the parent that died within the family was considered by bereaved parents as important to ensuring the children do not forget that parent. Integrated into everyday conversations, this included the sharing of memories about the parent that died, often facilitated by looking at photos and videos taken throughout the parent's life. Also, it was important for many bereaved parents that the children maintained a relationship with the family of the parent that died to aid their memory of the parent that died with cancer.

A connection to Paula’s [parent that died] family, I think that’s really important. The girls ask questions and the stories come to light about Paula which is a really good thing for creating a picture in their minds. And that brings me on to my biggest fear, my biggest fear is that the kids would forget her.[Sam, bereaved father]

There were some parents with cancer who wrote letters for their child's future before they died. Some bereaved parents considered it appropriate to give these to the children when they perceived the children to be ‘*mature enough to cherish them’.* In reality, it was considered as too painful by bereaved parents to give the children these letters written by the parent before they died. Alongside this, many bereaved parents felt it was inappropriate to give the children the letters, as often the ill‐parent died before they had time to finish them or write one for each of the children.

I still haven’t given it [letter] to the children. Again, I don’t know when the right time is to do that, how to do it. I thought in my own mind that I was going to have it all done and dusted for the first anniversary which has just passed. When it came to it, I felt it was too much for them to bare. And then I sort of had half an idea to do it over Easter but then I still thought it was too early. It’s over a year, and I feel as if I’m holding something back from them in not giving them this.[Oliver, bereaved father]

Fundraising for cancer charities that provide support to families impacted by parental cancer was described by many bereaved parents as an opportunity to honour the parent that died with cancer and facilitate quality time together as a family. Fundraising seemed important for bereaved parents to *‘give back’* to organisations that were helpful in the end of life or bereavement period. Also, to help families in the future who find themselves in these unfortunate situations.

It [referring to fundraising] brought me and the girls together. We were doing it for Ciara [parent that died]. It gave the girls the permission to talk about their mum. Fundraising brought us together, and at the same time knowing you’re giving for another family’s potential next week.[Michael, bereaved father]

## 4 DISCUSSION

Adapting to life after the death of a co‐parent with cancer is often very challenging for bereaved parents as they face the reality of parenting dependent children alone. Supported by the literature this included taking on the elements of parenting that were predominately led by the parent that died.^25^ However, this study provided new insights by identifying bereaved parents are uncertain about the appropriateness of being upset in front of the children regarding the death of the co‐parent with cancer. Alongside this, the timing of continuing with elements of life such as going back to work, keeping the memories alive of the parent who died and making new memories with the children such as going on holidays were highlighted as challenging by bereaved parents. Often, bereaved parents described their experience using metaphors which may have been in part to emphasise the unfortunate situation they have found themselves in, or helpful toward making sense of their own situation.^26^, ^27^

### 4.1 Clinical implications

Bereaved parents are central to supporting their children when a co‐parent has died with cancer.^11^ However, it is important that bereaved parents are looking after themselves so they can appropriately care for the children. It could be argued that bereaved parents struggled to adapt after the death of a co‐parent to cancer as they strived to provide more for their children than what they could practically and mentally offer.^28^, ^29^ If bereaved parents were encouraged to maximise social support networks to help with the practical elements of parenting and encouraged to practice self‐care this may aid a sense of equipoise as they navigate their own grief, as well as their children's.^28^

In general, it seemed the children adapted well after the death of a parent to cancer given that bereaved parents felt they coped better than anticipated. It could be suggested that the honest communication provided to the children regarding the parent's poor prognosis, provided throughout the end of life period, facilitated coping and adjustment in bereavement.^1^, ^3^ However, there were ongoing needs for the children of different developmental stages throughout bereavement. There is a need for bereaved parents to understand that grief is a process for children.^30^ Also, the reactions of children in bereavement may vary with some not responding in the way society suggests appropriate such as crying or yearning for their parent that died.^30^, ^31^ Bereaved parents should be reassured that it is alright to be upset in front of the children as this will provide the children with permission to share how they are feeling.^32^ This can create opportunities for the bereaved parents to provide the love and support that only a parent can provide.

Returning to school after the death of a parent can be a challenging time for the bereaved parent and children. School teachers play an instrumental role in the lives of children and appear important to facilitating a better bereavement experience for the family.^33^ Literature highlights professionals working in the school environment are uncertain about how best to support the bereaved children as well as their peers after a parent has died.^34^, ^35^ This necessitates a need for educational resources and guidance to be developed for these populations to support their provision of pastoral care to parentally bereaved families.

Keeping the memory alive of the parent that died with cancer was important to bereaved parents and the children. Similar findings have been reported in the literature.^36^ Findings highlight that bereaved parents struggled with the appropriate timing of providing the children with letters written by the parent with cancer before they died. It seemed most families cherished the photos and videos that were taken throughout the end of life period, emphasising the importance for parents to be encouraged to capture memories at the end of life period as they happen.^1^, ^3^

Meeting others who have experienced similar situations appeared to be powerful for bereaved parents facilitating hope for the here and now, and the future. It may be suggested peer‐support alleviates feelings of loneliness for bereaved parents as they now manage parenting alone. Also, literature highlights children value opportunities to meet and talk with other children who have experienced the death of a parent with cancer.^36^ Peer‐support has been reported as helpful toward promoting better mental and physical outcomes for people^37^; emphasising the importance of tailored peer‐support services for families when a parent of dependent children has died with cancer.

### 4.2 Study limitations

Findings are largely reflective of white two‐parent families where traditional roles of a mother and father are held. There is a need to better understand the experiences bereaved parents from a range of diverse, racial, ethnic, and cultural backgrounds, including those with diverse gender and sexual identifies. Although this study was conducted pre‐COVID‐19 pandemic, its findings are relevant regarding the needs of bereaved parents and children as professionals and services move forward. Also, similar findings have been highlighted during the COVID‐19 pandemic.^10^ As participation required individuals to respond to an invite or an advert, the needs of these individuals may differ to those who decided not to take part. This research was conducted in one region of the United Kingdom.

## 5 CONCLUSION

Despite bereaved parents being pivotal in navigating family life after the death of a co‐parent with cancer, this often presents many challenges as they confront this daunting new reality of parenting dependent children alone. In the abyss of loneliness and striving to be the perfect parent, it is important that bereaved parents are encouraged to practice self‐care and utilise support from social networks so they can appropriately meet the needs of their children. Soon after the death of a parent with cancer, family‐centred bereavement support groups should be available to provide both the bereaved parent and children with support and practical guidance. Peer‐support can normalise the grief experience and facilitate hope for the future.

## CONFLICT OF INTEREST

The authors declare that they have no conflict of interest.

## Supporting information

Supporting Information S1

## Data Availability

The data that supports the findings of this study are available at the Ulster University Repository and available on request from the first author. The data are not publicly available due to privacy and ethical restrictions. The study passed ethical committee review [REC:17/SW/0155].
